# Distribution of *sasX*, *mupA*, and *qacA/B* genes and determination of genetic relatedness of epidemic methicillin-resistant *Staphylococcus aureus* strains associated with bloodstream infections in southern China

**DOI:** 10.3389/fcimb.2025.1491658

**Published:** 2025-01-30

**Authors:** Rui Zhao, Bingyu Du, Lingling Hu, Chenxi Li, Fen Xue, Xing Wang, Changhong Jiang, Jinghua Wang, Yanfeng Zhao

**Affiliations:** ^1^ Clinical Microbiology Laboratory, Shanghai Center for Clinical Laboratory, Shanghai, China; ^2^ Laboratory Medicine Center, Second Affiliated Hospital, Nanjing Medical University, Nanjing, China; ^3^ Department of Laboratory Medicine, Shanghai Children’s Medical Center, Shanghai Jiao Tong University School of Medicine, Shanghai, China; ^4^ Department of Laboratory Medicine, Zhujiang Hospital, Southern Medical University, Guangzhou, China

**Keywords:** MRSA, MLST, bloodstream infections, *sasX*, *mupA*, *qacA/B*, sequence type

## Abstract

**Introduction:**

Methicillin-resistant *Staphylococcus aureus* (MRSA) exhibits diverse genotypes with varying virulence and resistance profiles, particularly in the context of bloodstream infections (BSI). This study investigates the prevalence of the *sasX*, *mupA*, and *qacA/B* genes among MRSA isolates from bloodstream infections in southern China and analyzes their genetic relatedness.

**Methods:**

A polymerase chain reaction (PCR) assay was developed to detect the presence of the *sasX* gene, which is associated with nasal colonization, immune evasion, and virulence, the mupirocin resistance gene *mupA*, and the chlorhexidine tolerance gene *qacA/B* in a total of 77 MRSA isolates. Multilocus sequence typing (MLST) was performed to determine the sequence types (STs) and assess the genetic relatedness of the isolates. The resistance of these strains to 16 antibiotics was also analyzed. The distribution of these genes and their association with epidemic STs were analyzed.

**Results:**

A total of 26 STs were identified, with notable prevalence in five epidemic clones: ST59, ST5, and ST764. The prevalence of the *sasX*, *mupA*, and qacA/B genes across all isolates was 23.4%, 33.8%, and 79.2%, respectively. Specifically, the frequency of the *sasX* gene was highest in ST59 (29.4%), ST239 (100%), and ST764 (37.5%); *mupA* was most prevalent in ST5 (66.7%), ST59 (17.6%), ST764 (37.5%), and ST15 (100%); *qacA/B* was predominantly found in ST59 (88.2%), ST5 (66.7%), ST398 (85.7%), ST764 (50.0%), and ST239 (100%). The gene distribution patterns revealed that *sasX+ qacA/B+ mupA+* strains were closely associated with epidemic clones ST6290 and ST88, whereas *sasX+ qacA/B+ mupA-* strains were linked to ST59, ST239, and ST764.

**Discussion:**

Notably, forty-seven (61%) MRSA BSI strains were multidrug-resistant, with the majority exhibiting resistance to penicillin, erythromycin, and clindamycin. Major MRSA clones in southern China include ST59, ST5, ST764, and ST398. In this study, *sasX*, *mupA* and *qacA/B* genes were present in the MRSA isolates, with the *mupA* gene being the most prevalent. Variations in the prevalence of virulence and resistance genes among these epidemic strains underscore the need for targeted infection control measures. These findings contribute to a better understanding of the genetic epidemiology of MRSA in the region, facilitating the development of effective prevention and control strategies for BSI.

## 1 Introduction

*Staphylococcus aureus*, a major Gram-positive coagulase-positive pathogen, is a spherical bacterium approximately 1 μm in diameter that forms grape-like clusters (Lakhundi and Zhang, 2018). This bacterium possesses a range of virulence factors and has the capability to develop resistance to most antibiotics and antiseptics, further compounded by the continual emergence of new clones. The clinical use of methicillin has led to the rise of methicillin-resistant *S. aureus* (MRSA). MRSA can adapt its genetic content and expression to generate new strains with enhanced virulence and colonization abilities. As a highly virulent and challenging pathogen, MRSA is a prevalent cause of both community-acquired and hospital-associated infections (Lakhundi and Zhang, 2018). Colonization with MRSA is a critical step in the pathogenesis of active infection and a key factor in the epidemiology of MRSA infections. Colonizing strains can act as endogenous reservoirs for overt clinical infections or spread to other patients, potentially leading to serious bacterial infections (Abad et al., 2013).

The novel *Staphylococcus aureus* cell wall-anchored protein gene, *sasX*, plays a critical role in enhancing nasal colonization, immune evasion, and overall virulence (Li et al., 2012). In addition to its role in nasal colonization, *sasX* is involved in biofilm formation and mechanisms of immune evasion. MRSA strains carrying *sasX* gene have been identified as potential causes of severe diseases, including pulmonary infections and abscess formation (Alam et al., 2022). The SasX protein, which is carried on the φSPβ-like prophage, was initially identified in the sequence type (ST) 239 TW20 isolated in the UK (Holden et al., 2010; Baines et al., 2015; Harris et al., 2010); However, *sasX* has also been detected in other STs, suggesting its association with diverse phenotypic expressions (Li et al., 2012; Holden et al., 2010; Wang et al., 2014).

Mupirocin, a topical antibiotic produced by *Pseudomonas fluorescens*, is used for the decolonization of both methicillin-susceptible *S. aureus* (MSSA) and MRSA in patients and healthcare personnel. It exhibits high efficacy against staphylococci, streptococci, and certain Gram-negative bacteria, including *Haemophilus influenzae* and *Neisseria gonorrhoeae* (Sutherland et al., 1985). Mupirocin is commonly employed to treat local skin and soft tissue infections caused by *S. aureus* and streptococcal species. Resistance to mupirocin is associated with the presence of the plasmid-encoded *mupA* gene, which is linked to high-level mupirocin resistance and therapeutic failure (Udo et al., 2001; Simor et al., 2007; Chaves et al., 2004; Cadilla et al., 2011). High-level mupirocin resistance, defined as a minimum inhibitory concentration (MIC) ≥512 mg/L, is mediated by the *mupA* gene that encodes an alternate isoleucyl-tRNA synthetase (IleRS-2) (Hetem et al., 2016), located on mobile genetic elements (Hetem and Bonten, 2013; Hodgson et al., 1994).

*Staphylococcus aureus* strains have shown decreased sensitivity to chlorhexidine, an antiseptic solution used globally since the 1950s (Milstone et al., 2008). This resistance is attributed to the presence of the *qacA/B* genes, which encodes proton-motive force-dependent export pumps (Mayer et al., 2001). The *qacA/B* genes are typically located on multiresistance plasmids and can co-exist with antimicrobial resistance genes, contributing to the persistence and survival of *qacA/B*-positive MRSA strains (Sidhu et al., 2002).The presence of the *qacA/B* genes is associated with elevated minimum bactericidal concentrations of chlorhexidine and failures in MRSA decolonization protocols (Warren et al., 2016).

Multilocus sequence typing (MLST) has been employed to study the genetic relatedness among MRSA isolates from clinical specimens. Among 119 European MRSA isolates, only two harbored *sasX*, and both were of the ST239 type (De Backer et al., 2019). MRSA ST239 is known for producing exotoxins that cause a range of severe infections (Abimanyu et al., 2012). The recent dissemination of *sasX* from ST239 to other invasive clones indicates that this ST shift may be a significant factor driving the MRSA epidemic in Asia (Li et al., 2012).

Detecting the *sasX*, *qacA/B*, and *mupA* genes, along with identifying circulating MRSA strains that carry these specific genes, has significant implications for nasal decolonization of *S. aureus*, reducing the infection rate, and guiding rational drug use in clinical practice. It provides crucial insights for optimizing therapeutic strategies and informs the development of more effective decontamination protocols currently in place.

## 2 Materials and methods

### 2.1 Collection and detection of MRSA isolates

The sample from three tertiary first-class hospitals, consisting of 77 non-duplicated convenience clinical MRSA isolates from patients in southern China collected between 2012 and 2020, provides a snapshot of the MRSA sequence type population in China. These strains were isolated from hospitalized patients with BSI. The identification of the MRSA isolates was performed based on colony morphology, antibiotic susceptibility testing, and *mec*A gene detection by PCR, as previously described (Kondo et al., 2007). Bacterial identification and oxacillin resistance (susceptibility testing) were performed using the Vitek 2 Compact Automated Microbiology System (BioMérieux, Durham, NC, USA) following the manufacturer’s instructions in a clinical microbiology laboratory. A total of 16 drugs were tested: penicillin, erythromycin, oxacillin, clindamycin, ciprofloxacin, moxifoxacin, levofloxacin, tetracycline, gentamicin, rifampicin, trimethoprim–sulfamethoxazole, quinupristin/dalfopristin, linezolid, tigecycline, vancomycin and cefoxitin. Results were interpreted according to the recommendations and definitions of the Clinical and Laboratory Standards Institute [CLSI]. *S. aureus* ATCC 29213 was used for quality control in antimicrobial susceptibility testing. All MRSA strains were stored at −80°C until use.

### 2.2 Genomic DNA extraction

All isolates were cultured on blood agar (CHROMagar) and incubated overnight at 37°C. Bacterial DNA was isolated using a TIANamp Bacteria DNA Kit (TIANGEN, Beijing, China) according to the manufacturer’s instructions and used as the template for all PCRs.

### 2.3 Gene typing

MLST was performed on all isolates by amplifying and sequencing seven internal housekeeping gene fragments—*arcC*, *aroE*, *glpF*, *gmk*, *pta*, *tpi*, and *yqi*—each approximately 450 bp in length (Enright et al., 2000), using the Mastercycler Nexus Gradient Thermal Cycler (Eppendorf). The sequence profile and ST of each allele were determined according to the MLST database (http://saureus.mlst.net). The allelic profiles were assigned by comparing the sequences at each locus with those of the known alleles in the *S. aureus* MLST database, and the profiles were then defined as the STs. Each sequence contig was submitted to the *Staphylococcus aureus* webpage on PubMLST (https://pubmlst.org/organisms/staphylococcus-aureus) for allelic profiles and ST characterization. In our study, primers were designed manually and obtained commercially. All oligonucleotide primers used were synthesized by Sangon Biotech (Shanghai, China).

### 2.4 Analysis of phylogenetic

GrapeTree (Zhou et al., 2018) implements a novel minimum spanning tree algorithm (MSTree V2) to reconstruct genetic relationships from allelic profiles as a minimum spanning tree. Distances between isolates are calculated based on the number of shared multilocus sequence typing (MLST) alleles. Additionally, the Interactive Tree of Life (iTOL) (Letunic and Bork, 2021) generates neighbor-joining phylogenies for visualization, incorporating annotations for virulence factors, MLST, and the geographic locations of isolates. Both the GrapeTree and iTOL plugins are hosted on the PubMLST website (https://pubmlst.org/).

### 2.5 PCR assay for gene detection

Detection of the *sasX*, *mupA* and *qacA/B* genes was conducted using published protocols (Monecke et al., 2014; Noguchi et al., 2005; Yun et al., 2003).The specific primers used for the identification of the respective targets —*sasX*, *mupA*, and *qacA/B* genes are listed in **Table 1**.

**Table 1:** List of primer sequences for each target gene.

Target gene	Primer set	Primer sequence(5’ to 3’ end)
*mecA*	*mecA-F*	GCCGTAGTTGTCGGGTTTGG
	*mecA-R*	GGCGGATGTGCGATTGTATTGC
*sasX*	*sasX-F*	AGAATTAGAAGTACGTCTAAATGC
	*sasX-R*	GCTGATTATGTAAATGACTCAAATG
*mupA*	*mupA-F*	CATTGGAAGATGAAATGCATACC
	*mupA-R*	CGCAGTCATTATCTTCACTGAG
*qacA/B*	*qacA/B-F*	CTATGGCAATAGGAGATATGGTGT
	*qacA/B-R*	CCACTACAGATTCTTCAGCTACATG

The *sasX*, *mupA*, and *qacA/B* genes were amplified using a S1000 thermal cycler (Bio-Rad, Hercules, California, USA) under the following conditions: 5 minutes of denaturation at 94°C, followed by 32 cycles consisting of 45 seconds at 94°C, 30 seconds at 56°C, and 1 minute at 72°C, with a final 7-minute elongation step at 72°C. The amplified PCR products were analyzed by agarose gel electrophoresis on a 1.2% gel in 1×TAE buffer at 110V for 42 minutes, then stained with ethidium bromide and exposed to UV light for visualization. If the bands were not completely clear, the experiment was repeated to confirm reproducibility. The gel was also stained with Florosafe (Apical Scientific Sdn Bhd, Selangor, Malaysia) and visualized using a gel imaging system (AlphaImager; Alpha Innotec, Kasendorf, Germany). The PCR products were sent for sequencing analysis (Apical Scientific Sdn Bhd, Selangor, Malaysia), which was performed bidirectionally. In addition to the genes associated with MRSA decolonization protocols, we also tested adhesion genes (*sdrC*, *sdrD*, *sdrE, icaA*, and *clfA*), as previously described (Peacock et al., 2002; Campbell et al., 2008). All primers are listed in **Supplementary Table S2**.

### 2.6 Statistical analysis

Statistical analyses were performed using GraphPad Prism 9.0 (GraphPad Software Inc.; San Diego, CA, USA) and IBM SPSS Statistics (SPSS Inc., Chicago, IL, USA). All graphs related to the study were constructed and described in terms of percentages and frequencies. Pearson’s chi-square test or Fisher’s exact test was used to determine whether differences in the frequency of *sasX*, *mupA*, and *qacA/B* genes exist among isolates with different ST types. Statistical significance was set at a *P-value* of <0.05. The descriptive data were presented as percentages for the categorical data.

## 3 Results

A total of 77 MRSA isolates were analyzed, revealing the presence of *sasX*, *mupA*, and *qacA/B* genes across various STs, as detailed in **Table 2**. PCR results indicated that 18 isolates (23.4%) were positive for the *sasX* gene, 26 isolates (33.8%) for the *mupA* gene, and 61 isolates (79.2%) for the *qacA/B* genes. The predominant STs with *sasX* were ST59 (5 isolates, 6.5%), ST239 (3 isolates, 3.9%), and ST764 (3 isolates, 3.9%). For *mupA*, the most common STs were ST5 (6 isolates, 7.8%), ST59 (3 isolates, 3.9%), and ST764 (3 isolates, 3.9%). The most frequent STs for *qacA/B* were ST59 (15 isolates, 19.5%), ST5 (6 isolates, 7.8%), ST398 (6 isolates, 7.8%), and ST764 (4 isolates, 5.2%). Co-existence of *sasX* and *qacA/B* genes was observed exclusively in 6.5% (n = 5) of MRSA ST59, and in 3.9% (n = 3) of MRSA ST239 and ST764. The simultaneous presence of *sasX*, *qacA/B*, and *mupA* genes occurred in 1.3% (n = 1) of MRSA ST6290 and 1.3% (n = 1) of MRSA ST88 (see **Table 3**).

**Table 2:** Frequency of *sasX*, *qacA/B*, and *mupA* genes detection in 77 MRSA isolates.

Genes	MRSA(n,%)	MLST(n,%)
*sasX*	18, 23.4%	ST59(5,6.5%),ST239(3,3.9%),ST764(3,3.9%),ST88(2,2.6%),ST6290(1,1.3%),ST630(1,1.3%),ST398(1,1.3%),ST5(1,1.3%),ST7212(1,1.3%)
*mupA*	26, 33.8%	ST5(6,7.8%),ST59(3,3.9%),ST764(3,3.9%),ST15(2,2.6%),ST951(2,2.6%),ST6697(2,2.6%),ST398(2,2.6%),ST22(1,1.3%),ST88(1,1.3%),ST6570(1,1.3%),ST6290(1,1.3%),ST5985(1,1.3%),ST88(1,1.3%)
*qacA/B*	61, 79.2%	ST59(15,19.5%),ST5(6,7.8%),ST398(6,7.8%),ST764(4,5.2%),ST239(3,3.9%),ST1(3,3.9%),ST88(3,3.9%),ST951(3,3.9%),ST45(2,2.6%),ST15(2,2.6%),ST6290(2,2.6%),ST22(1,1.3%),ST188(1,1.3%),ST25(1,1.3%),ST30(1,1.3%),ST338(1,1.3%),ST5985(1,1.3%),ST6285(1,1.3%),ST6570(1,1.3%),ST30(1,1.3%),ST6697(1,1.3%),ST72(1,1.3%),ST7212(1,1.3%)

**Table 3:** Distinctive sequence types with gene distribution patterns of 77 MRSA isolates.

Pattern of genes	MLST(n,%)
*sasX+ qacA/B+ mupA+*	ST6290(1,1.3%),ST88 (1,1.3%)
*sasX+ qacA/B+ mupA-*	ST59(5,6.5%),ST239(3,3.9%),ST764(3,3.9%),ST7212(1,1.3%),ST630(1,1.3%),ST88(1,1.3%),ST398(1,1.3%),ST5(1,1.3%)
*sasX- qacA/B- mupA-*	ST59(1,1.3%),ST1(1,1.3%),ST546(1,1.3%),ST6(1,1.3%),ST338(1,1.3%),ST764(1,1.3%),ST5904(1,1.3%)

**Figure 1:**
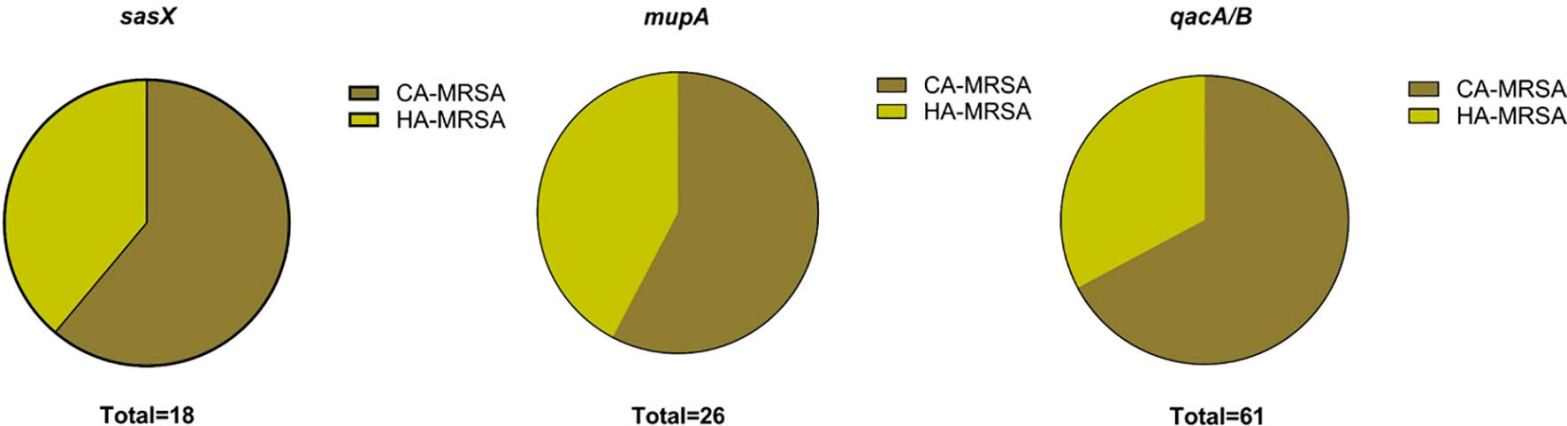
Charts show the distribution of HA-MRSA and CA-MRSA based on the presence of the respective target genes: *sasX*, *mupA*, and *qacA/B*.

**Figure 2:**
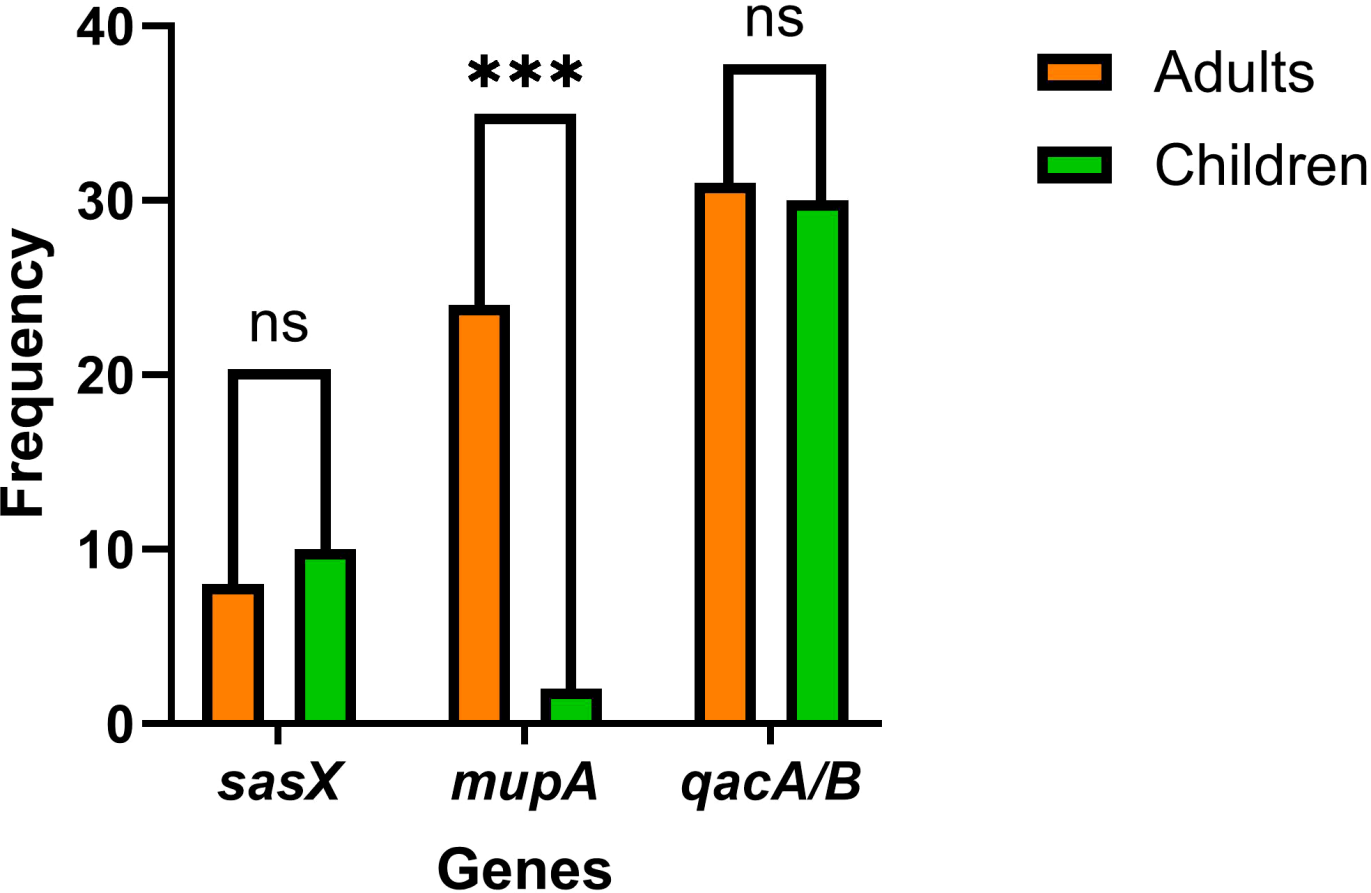
The graph shows the frequencies of *sasX*, *qacA/B*, and *mupA* genes in MRSA isolates from adults and children. *** indicates *p* < 0.001, and “ns (no significance)”, indicates *p* > 0.05.

The proportional distribution of CA-MRSA and HA-MRSA strains across various specimens is shown in **Figure 1**. No significant differences were observed in the detection rates of individual genes between hospital-acquired and community-acquired MRSA. The detection frequencies of the three genes in adults and children are presented in **Figure 2**, where the detection rate of *mupA* in adults was higher than that in children (*P* < 0.001).

The *icaA* and *clfA* genes were the most frequently detected adhesion genes, with detection rates of 100% and 96.1%, respectively. However, the detection rate of *sdrD* was only 64.9% (**Supplementary Table S1**). The *sdrC*, *sdrD*, *sdrE*, *icaA*, and *clfA* genes were detected in all ST1-MRSA isolates and most ST5-MRSA isolates. Relatively low detection rates of *sdrD* were observed among ST59-MRSA and ST398-MRSA isolates. Only two MRSA isolates from ST398 harbored the *sdrE* gene, as shown in **Supplementary Table S1**.

The antimicrobial resistance profiles of 77 *S. aureus* isolates are displayed in **Supplementary Figure S1**. All strains were resistant to penicillin, oxacillin, and cefoxitin, but fully susceptible to several tested antibiotics, including quinupristin/dalfopristin, linezolid, vancomycin, and tigecycline. Notably, forty-seven (61%) *S. aureus* BSI strains were multidrug-resistant, with the majority being resistant to penicillin, oxacillin, erythromycin, and clindamycin.

**Figure 3:**
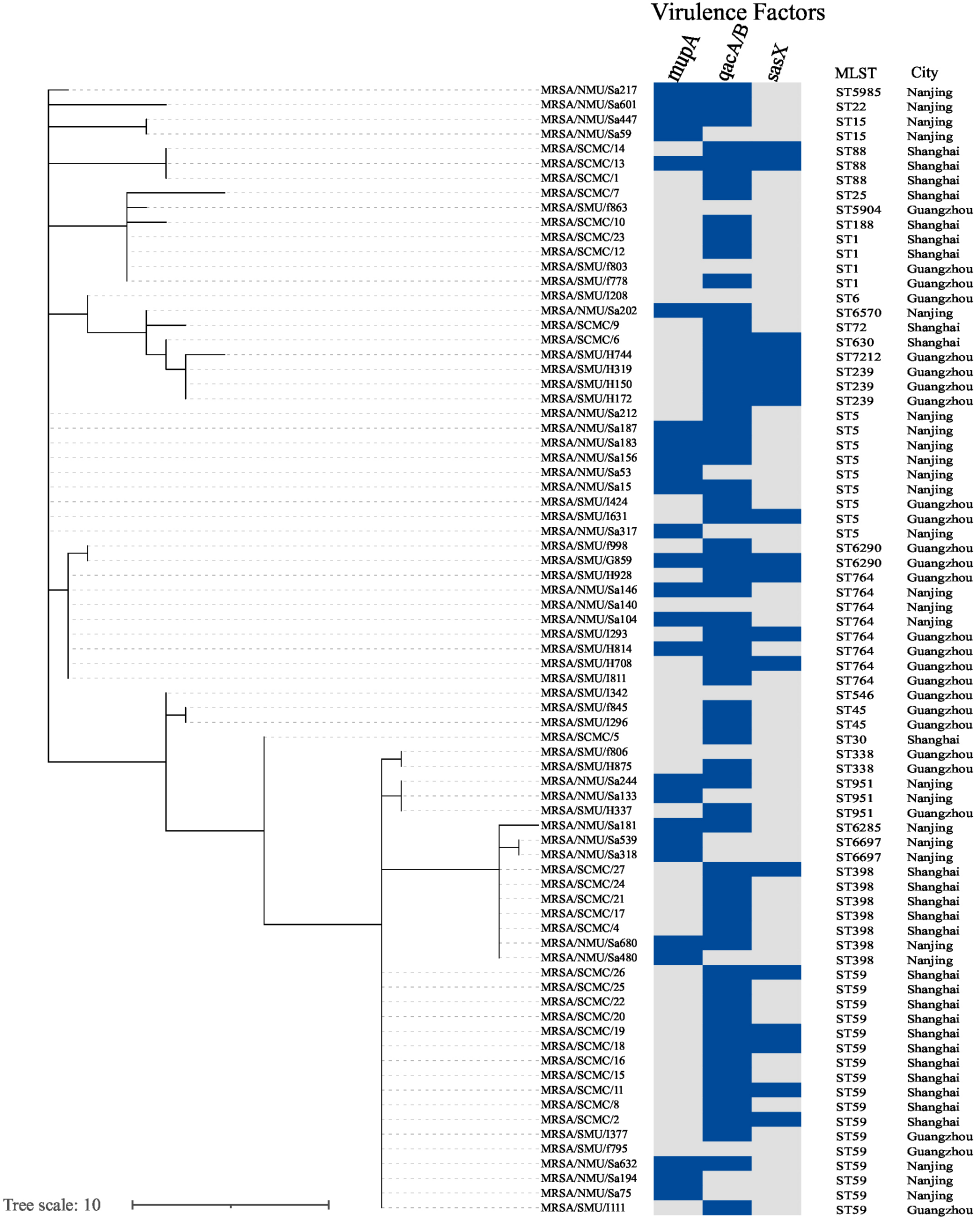
Phylogenetic tree shows the genetic relatedness of isolates (n = 77). Phylogenetic tree inferred by MSTree V2. The tree was constructed using MLST reference genomes (n = 77) and was labeled with the metadata using iTOL v.6. Columns represent, respectively, the isolate ID, sequence type (ST), hospital city location. The presence of various antimicrobial resistance genes in the genomes is indicated as a blue box.

Overall, 26 STs were identified among the MRSA strains. Phylogenetic analysis revealed significant genetic variability among the MRSA isolates, as shown in **Figure 3**. Three predominant clones—ST59, ST5, and ST764—were identified. The phylogenetic tree demonstrated that MRSA isolates obtained from clinical blood culture samples were clonal. The phylogram, reflecting the evolutionary relationships among clinical epidemic MRSA isolates from southern China (n = 77), is based on MLST homology (**Figure 3**).

## 4 Discussion

MRSA remains one of the most successful contemporary pathogens, demonstrating remarkable adaptability as both a commensal organism and a leading cause of severe infections in healthcare and community settings. It is responsible for a broad spectrum of clinical manifestations, including bacteremia, endocarditis, skin and soft tissue infections, bone and joint infections, and hospital-acquired infections (Turner et al., 2019). MRSA colonization significantly increases the risk of developing ventilator-associated pneumonia following intubation, particularly in intensive care units, and is associated with elevated mortality rates among hospitalized patients (Percival et al., 2015). Patients who have MRSA nasal colonization are at high risk of developing subsequent infections. The epidemiology of MRSA is characterized by the serial emergence of epidemic strains, posing a persistent clinical threat with consistently high morbidity and mortality rates (Turner et al., 2019). Analyzing the distribution of *sasX*, *qacA/B*, and *mupA* genes in MRSA clones is crucial for understanding the pathogen’s evolution and dissemination across different regions.

The *sasX* gene, although rarely detected in clinical strains, has been identified at varying frequencies across different studies. Nakaminami et al. reported a *sasX* prevalence of only 0.3% among isolated strains (Nakaminami et al., 2017), while Kong et al. detected it in 5.8% of their isolates (Kong et al., 2018). Surprisingly, *sasX* was relatively frequently identified in the study by Zieliński W et al., with 69% of *sasX*-positive isolates (75 out of 108) being *S. aureus*. A study conducted in a Malaysian hospital detected the *sasX* gene in 14.9% of cases (Alam et al., 2022), with one case involving ST4649 and the remainder (n = 13) exclusively from ST239 (Alam et al., 2022). This finding is consistent with a previous study (Zarizal et al., 2018). Another study conducted in a Chinese hospital found *sasX* in 36.7% of cases (Song et al., 2013). Despite the common association of the *sasX* gene with ST239, Nair et al. reported in 2013 that the *sasX* gene was not detected in any MRSA isolates, including those of ST239, which may reflect its low prevalence in the Mongolian hospital studied (Nair et al., 2013). Moreover, recent evidence suggests that *sasX* has spread from ST239 to other invasive clones across different sequence types (Li et al., 2012). In our study, 23.4% (n = 18) of MRSA isolates carried the *sasX* gene. Among these, five were from ST59 (5/17, 29.4%), three from ST239 (3/3, 100%), and three from ST764 (3/8, 37.5%) strains. Notably, all three ST239 strains in this study contained the *sasX* gene, making ST239 the dominant lineage among *sasX*-positive isolates, with a 100% detection rate within this sequence type. The *sasX* gene, a colonization-virulence factor, likely contributes to the success of MRSA ST239 in Asia (De Backer et al., 2019). Our data underscore the dissemination of *sasX* to non-ST239 sequence types, highlighting its broader impact on MRSA epidemiology.

Additionally, antiseptic resistance genes *qacA/B* were detected in 79.2% (n = 61) of MRSA isolates in our study. This finding aligns with the report by Shamsudin MN et al., who in 2012 observed a high prevalence of *qacA/B*-positive MRSA at 83.3% (50 out of 60) in Malaysian isolates (Shamsudin et al., 2012). However, a more recent study from Malaysia reported a much lower prevalence, with *qacA/B* genes detected in only 7.4% (n = 7) of MRSA isolates. These findings, including those from our study and others, suggest significant regional differences in the prevalence of *qacA/B* genes among MRSA isolates. Our results also indicate a notable association between *qacA/B* genes and specific sequence types. For example, *qacA/B* genes were frequently present in ST59 (15/17, 88.2%), ST5 (6/9, 66.7%), ST398 (6/7, 85.7%), ST764 (4/8, 50.0%), and ST239 (3/3, 100%) isolates. This observation is consistent with a report by Lu et al., who found that 88.0% (22/25) of ST239 MRSA isolates were *qacA/B*-positive (Lu et al., 2015). Similarly, Ho et al. reported a high frequency of *qacA/B* genes (88.9%) in ST239 isolates (Ho and Branley, 2012). Conversely, Kong H et al. observed that the highest incidence of *qacA/B* genes was in ST5 clones (34.1%), which were the predominant clone in their MRSA isolates from the region (Kong et al., 2018). In Taiwan, several studies screening MRSA isolates from various hospitals over different periods consistently found that the genotypic resistance rate to chlorhexidine, attributed to the presence of *qacA/B* genes, ranged from 35.4% to 55.4% (Sheng et al., 2009; Wang et al., 2008; Lee et al., 2022). Among different sequence types, ST239 MRSA isolates exhibited the highest resistance to both chlorhexidine and other antimicrobial agents (Sheng et al., 2009; Lee et al., 2022), likely due to the widespread use of chlorhexidine in hospital settings (Wang et al., 2008).

Previous studies have also reported an increased incidence of mupirocin resistance in MRSA, leading to the failure of decolonization treatments (Lu et al., 2015). Several MRSA decolonization studies have shown that 6.9%–10.9% of MRSA isolates were mupirocin-resistant, and all carried the *mupA* gene (Warren et al., 2016; Hayden et al., 2016; Muñoz-Gallego et al., 2016). Notably, no mupirocin-resistant MRSA isolates were reported in Taiwan before 2010 (Chen et al., 2014); however, by later years, 91.5% of MRSA isolates harbored the *mupA* gene, despite mupirocin not being routinely used for MRSA decolonization in the country (Lee et al., 2022).

In our study, the *mupA* gene was detected in 26 (33.8%) of the MRSA isolates. This gene was predominantly found in ST5 (6/9, 66.7%), ST59 (3/17, 17.6%), ST764 (3/8, 37.5%), and ST15 (2/2, 100%) isolates. Notably, all ST15 isolates exhibited a 100% detection rate for the *mupA* gene, whereas none of the ST239 isolates harbored the *mupA* gene. This contrasts with an earlier Malaysian study, which reported that 70% (11 out of 16) of ST239 isolates were positive for the *mupA* gene (Ghasemzadeh-Moghaddam et al., 2014). In contrast, studies by Alam NNNB et al. and Nejabat et al. found no *mupA* gene presence in any of their MRSA isolates (Alam et al., 2022). Our study underscores the importance of MLST data analysis in assessing the genetic variability of MRSA isolates, investigating the association between the *sasX*, *qacA/B*, and *mupA* genes with different STs, and examining the genetic relatedness among MRSA strains from different regions. Notably, this study revealed the co-existence of the *sasX*, *qacA/B*, and *mupA* genes in MRSA isolates, with this gene co-existence occurring exclusively in ST6290 and ST88 isolates. The gene distribution pattern from all of the isolates showed that *sasX+ qacA/B+ mupA-* was closely associated with epidemic clones ST59, ST239, and ST764. Both *sasX* and *qacA/B* genes were detected in ST239, consistent with previous studies (Alam et al., 2022; Song et al., 2013).

Obtaining relevant information about the *sasX*, *qacA/B*, and *mupA* genes is greatly helpful for nasal decolonization of *S. aureus*, reducing the infection rate, and guiding rational drug use in clinical practice. The involvement of adhesion genes is also important in contributing to the severity of infections and the pathogen’s endemicity. Adhesion genes were detected in the majority of MRSA isolates. The positivity rates for the *icaA and clfA* genes were high in our study, and all ST1-MRSA isolates carried the five genes with a 100% detection rate, which were consistent with previous findings (Wang et al., 2018; Foster et al., 2020). However, some findings differ from previous reports. For example, the *icaA* gene was detected in all isolates except for ST188 (Liu et al., 2023). Only 16.7% (8/48) of isolates harbored the *icaA* gene (Zamani et al., 2022). The carrying rates for the *sdrC*, *sdrD*, and *sdrE* genes were 76.7%, 20.0%, and 91.7%, respectively (Yang et al., 2017).Phylogenetic analysis revealed genetic diversity among MRSA isolates. The most common MRSA clones in southern China were ST59, ST5, ST764, and ST398, as identified from blood samples. Notably, 47 (61%) MRSA BSI strains were multidrug-resistant, with the highest resistance observed against penicillin, erythromycin, and clindamycin. These findings are consistent with those reported in previous studies (Wang et al., 2018).

In clinical practice, one of the critical hygienic measures to prevent the spread of *S. aureus* is the decontamination of potentially contaminated rooms, utensils, and colonized patients. Therefore, the isolation of *S. aureus* strains carrying antiseptic resistance genes from clinical samples is concerning (Kong et al., 2018). Our findings could be instrumental in developing more effective control and prevention strategies for nosocomial MRSA infections. However, this study has limitations. We did not perform a correlation validation between laboratory results and clinical outcomes, which would be a valuable direction for future research. Additionally, differences in study approaches and sample sizes limit the comparability of our data with that of other studies.

## 5 Conclusion

In conclusion, we analyzed a total of 77 MRSA isolates from a healthcare setting, revealing the presence of the *sasX*, *mupA*, and *qacA/B* genes, which are associated with increased mortality rates in hospitalized patients. The detection rates of these genes across all isolates were 23.4%, 33.8%, and 79.2%, respectively. Our findings demonstrated that circulating MRSA genotypes exhibit varying virulence and resistance determinants, with significant differences in gene prevalence among STs. Notably, ST59 (5/17), ST239 (3/3), and ST764 (3/8) isolates had the highest prevalence of *sasX*, while ST59, ST398, and ST239 showed the highest rates of *qacA/B*. Additionally, ST5 and ST15 isolates exhibited higher incidences of the *mupA* gene compared to other STs. Despite these findings, our understanding of the distribution and associations of *sasX*, *qacA/B*, and *mupA* genes in MRSA isolates remains limited, highlighting the need for ongoing, intensive research. Continued studies are crucial to further elucidate the genetic diversity, evolution, and epidemiology of MRSA.

## Data Availability

The original contributions presented in the study are included in the article/**Supplementary Material**. Further inquiries can be directed to the corresponding authors.
