# Supplementary material for: Distribution of sasX, mupA, and qacA/B genes and determination of genetic relatedness of epidemic methicillin-resistant Staphylococcus aureus strains associated with bloodstream infections in southern China
Source: Front Cell Infect Microbiol. 2025 Jan 30;15:1491658. doi: 10.3389/fcimb.2025.1491658 (PMC11821609; doi:10.3389/fcimb.2025.1491658)
Supplement: Supplementary file 1 [file DataSheet1.pdf]

Supplementary Tables

Table S1 Frequencies and distribution of sequence types of *sdrC*, *sdrD*, *sdrE*, *icaA*, and *clfA* genes among 77 MRSA isolates.

Genes	(n, %[a])	MLST（n, %[b]）
sdrC	70,90.9	ST59(16,94.1),ST5(9,100),ST764(5,62.5),ST398(7,100),ST1(4,100),ST239(2,66.7),ST951(3,100),ST88(3,100),ST45(1,50),ST338(2,100),ST15(2,100),ST6290(2,100),ST6697(2,100),ST6(1,100),ST5985(1,100),ST6570(1,100),ST630(1,100),ST6285(1,100),ST5904(1,100),ST22(1,100),ST25(1,100),ST30(1,100),ST546(1,100),ST72(1,100),ST188(1,100)
sdrD	50,64.9	ST59(6,35.3),ST5(9,100),ST764(5,62.5),ST398(2,28.6),ST1(4,100),ST239(3,100),ST951(3,100),ST88(2,66.7),ST45(1,50),ST338(1,50),ST15(2,100),ST6290(2,100),ST6697(2,100),ST6(1,100),ST5985(1,100),ST6570(1,100),ST6285(1,100),ST5904(1,100),ST22(1,100),ST25(1,100),ST72(1,100)
sdrE	64,83.1	ST59(16,94.1),ST5(9,100),ST764(7,87.5),ST398(2,28.6),ST1(4,100),ST239(3,100),ST951(2,66.7),ST88(2,66.7),ST45(1,50),ST338(2,100),ST15(2,100),ST6290(2,100),ST6697(2,100),ST6(1,100),ST5985(1,100),ST6570(1,100),ST7212(1,100),ST6285(1,100),ST5904(1,100),ST22(1,100),ST25(1,100),ST546(1,100),ST188(1,100)
clfA	74,96.1	ST59(17,100),ST5(8,88.9),ST764(7,87.5),ST398(7,100),ST1(4,100),ST239(3,100),ST951(3,100),ST88(3,100),ST45(2,100),ST338(2,100),ST15(2,100),ST6290(2,100),ST6697(2,100),ST6(1,100),ST5985(1,100),ST7212(1,100),ST630(1,100),ST6285(1,100),ST5904(1,100),ST22(1,100),ST25(1,100),ST30(1,100),ST546(1,100),ST72(1,100),ST188(1,100)
icaA	77,100	ST59(17,100),ST5(9,100),ST764(8,100),ST398(7,100),ST1(4,100),ST239(3,100),ST951(3,100),ST88(3,100),ST45(2,100),ST338(2,100),ST15(2,100),ST6290(2,100),ST6697(2,100),ST6(1,100),ST5985(1,100),ST6570(1,100),ST7212(1,100),ST630(1,100),ST6285(1,100),ST5904(1,100),ST22(1,100),ST25(1,100),ST30(1,100),ST546(1,100),ST72(1,100),ST188(1,100)

[a]The positive rates of genes among 77 MRSA isolates. [b]The positive rates of *sdrC, sdrD, sdrE, icaA,* and *clfA genes* in different sequence types.

Table S2 Primers for *sdrC, sdrD, sdrE, icaA,* and *clfA* genes used in the study.

Target gene	Primer set	Primer sequence(5' to 3' end)
sdrC	*sdrC-F*	ACGACTATTAAACCAAGAAC
	sdrC-R	GTACTTGAAATAAGCGGTTG
sdrD	*sdrD-F*	GGAAATAAAGTTGAAGTTTC

	sdrD-R	ACTTTGTCATCAACTGTAAT
sdrE	*sdrE-F*	CAGTAAATGTGTCAAAAGA
	sdrE-R	TTGACTACCAGCTATATC
icaA	*icaA-F*	GATTATGTAATGTGCTTGGA
	icaA-R	ACTACTGCTGCGTTAATAAT
clfA	*clfA-F*	GTAGGTACGTTAATCGGTT
	clfA-R	CTCATCAGGTTGTTCAGG

Supplementary Figure

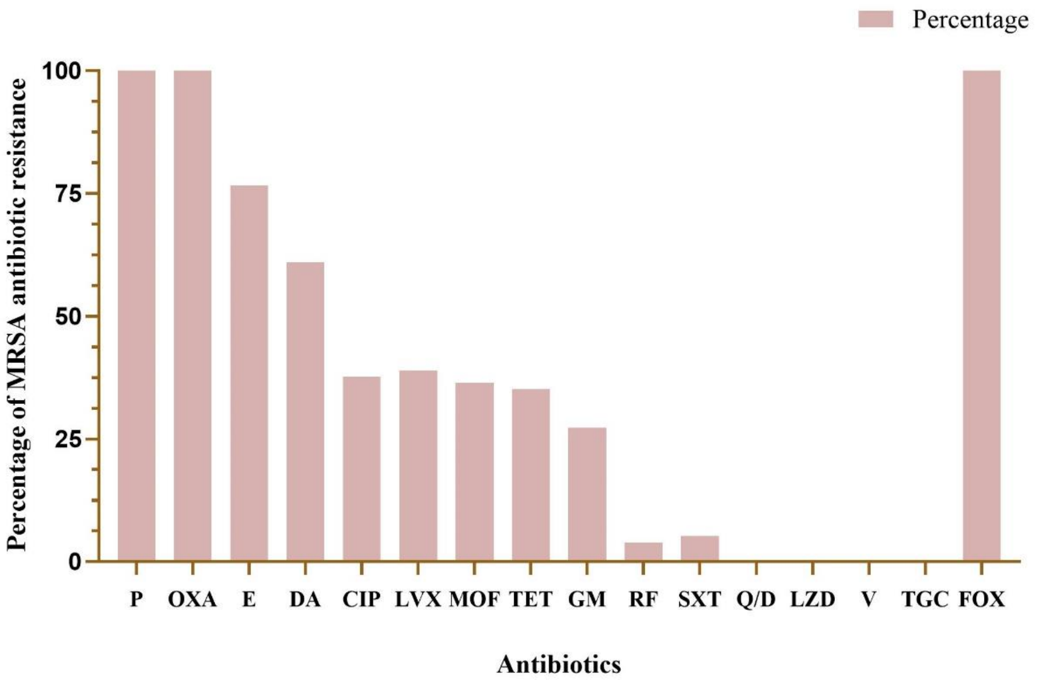

Supplementary Figure 1. Antimicrobial resistance profiles of MRSA isolates from adult and pediatric patients. Abbreviations: P, penicillin; OXA, oxacillin; E, erythromycin; DA, clindamycin; CIP, ciprofloxacin; LVX, levofloxacin; MOF, moxifloxacin; TET, tetracycline; GM, gentamicin; RF, rifampicin; SXT, trimethoprim-sulfamethoxazole; Q/D, quinupristin/dalfopristin; LZD, linezolid; V, vancomycin; TGC, tigecycline; FOX, cefoxitin.
