# Supplementary material for: Distribution of sasX, mupA, and qacA/B genes and determination of genetic relatedness of epidemic methicillin-resistant Staphylococcus aureus strains associated with bloodstream infections in southern China
Source: Front Cell Infect Microbiol. 2025 Jan 30;15:1491658. doi: 10.3389/fcimb.2025.1491658 (PMC11821609; doi:10.3389/fcimb.2025.1491658)
Supplement: Supplementary file 2 [file Table1.docx]

**Supplementary Tables**

**Table S1** Frequencies and distribution of sequence types of *sdrC*, *sdrD*, *sdrE*, *icaA*, and *clfA* genes among 77 MRSA isolates.

| Genes | (n, %^a^) | MLST（n, %^b^） |
| --- | --- | --- |
| *sdrC* | 70,90.9 | ST59(16,94.1),ST5(9,100),ST764(5,62.5),ST398(7,100),ST1(4,100),ST239(2,66.7),ST951(3,100),ST88(3,100),ST45(1,50),ST338(2,100),ST15(2,100),ST6290(2,100),ST6697(2,100),ST6(1,100),ST5985(1,100),ST6570(1,100),ST630(1,100),ST6285(1,100),ST5904(1,100),ST22(1,100),ST25(1,100),ST30(1,100),ST546(1,100),ST72(1,100),ST188(1,100) |
| *sdrD* | 50,64.9 | ST59(6,35.3),ST5(9,100),ST764(5,62.5),ST398(2,28.6),ST1(4,100),ST239(3,100),ST951(3,100),ST88(2,66.7),ST45(1,50),ST338(1,50),ST15(2,100),ST6290(2,100),ST6697(2,100),ST6(1,100),ST5985(1,100),ST6570(1,100),ST6285(1,100),ST5904(1,100),ST22(1,100),ST25(1,100),ST72(1,100) |
| *sdrE* | 64,83.1 | ST59(16,94.1),ST5(9,100),ST764(7,87.5),ST398(2,28.6),ST1(4,100),ST239(3,100),ST951(2,66.7),ST88(2,66.7),ST45(1,50),ST338(2,100),ST15(2,100),ST6290(2,100),ST6697(2,100),ST6(1,100),ST5985(1,100),ST6570(1,100),ST7212(1,100),ST6285(1,100),ST5904(1,100),ST22(1,100),ST25(1,100),ST546(1,100),ST188(1,100) |
| *clfA* | 74,96.1 | ST59(17,100),ST5(8,88.9),ST764(7,87.5),ST398(7,100),ST1(4,100),ST239(3,100),ST951(3,100),ST88(3,100),ST45(2,100),ST338(2,100),ST15(2,100),ST6290(2,100),ST6697(2,100),ST6(1,100),ST5985(1,100),ST7212(1,100),ST630(1,100),ST6285(1,100),ST5904(1,100),ST22(1,100),ST25(1,100),ST30(1,100),ST546(1,100),ST72(1,100),ST188(1,100) |
| *icaA* | 77,100 | ST59(17,100),ST5(9,100),ST764(8,100),ST398(7,100),ST1(4,100),ST239(3,100),ST951(3,100),ST88(3,100),ST45(2,100),ST338(2,100),ST15(2,100),ST6290(2,100),ST6697(2,100),ST6(1,100),ST5985(1,100),ST6570(1,100),ST7212(1,100),ST630(1,100),ST6285(1,100),ST5904(1,100),ST22(1,100),ST25(1,100),ST30(1,100),ST546(1,100),ST72(1,100),ST188(1,100) |

^a^The positive rates of genes among 77 MRSA isolates. ^b^The positive rates of *sdrC*, *sdrD*, *sdrE*, *icaA*, and *clfA genes* in different sequence types.

**Table S2** Primers for *sdrC*, *sdrD*, *sdrE*, *icaA*, and *clfA* genes used in the study.

| Target gene | | Primer set | Primer sequence(5’ to 3’ end) |
| --- | --- | --- | --- |
| *sdrC* | *sdrC-F* | | ACGACTATTAAACCAAGAAC |
|  | *sdrC-R* | | GTACTTGAAATAAGCGGTTG |
| *sdrD* | *sdrD-F* | | GGAAATAAAGTTGAAGTTTC |
|  | *sdrD-R* | | ACTTTGTCATCAACTGTAAT |
| *sdrE* | *sdrE-F* | | CAGTAAATGTGTCAAAAGA |
|  | *sdrE-R* | | TTGACTACCAGCTATATC |
| *icaA* | *icaA-F* | | GATTATGTAATGTGCTTGGA |
|  | *icaA-R* | | ACTACTGCTGCGTTAATAAT |
| *clfA* | *clfA-F* | | GTAGGTACGTTAATCGGTT |
|  | *clfA-R* | | CTCATCAGGTTGTTCAGG |

**Supplementary Figure
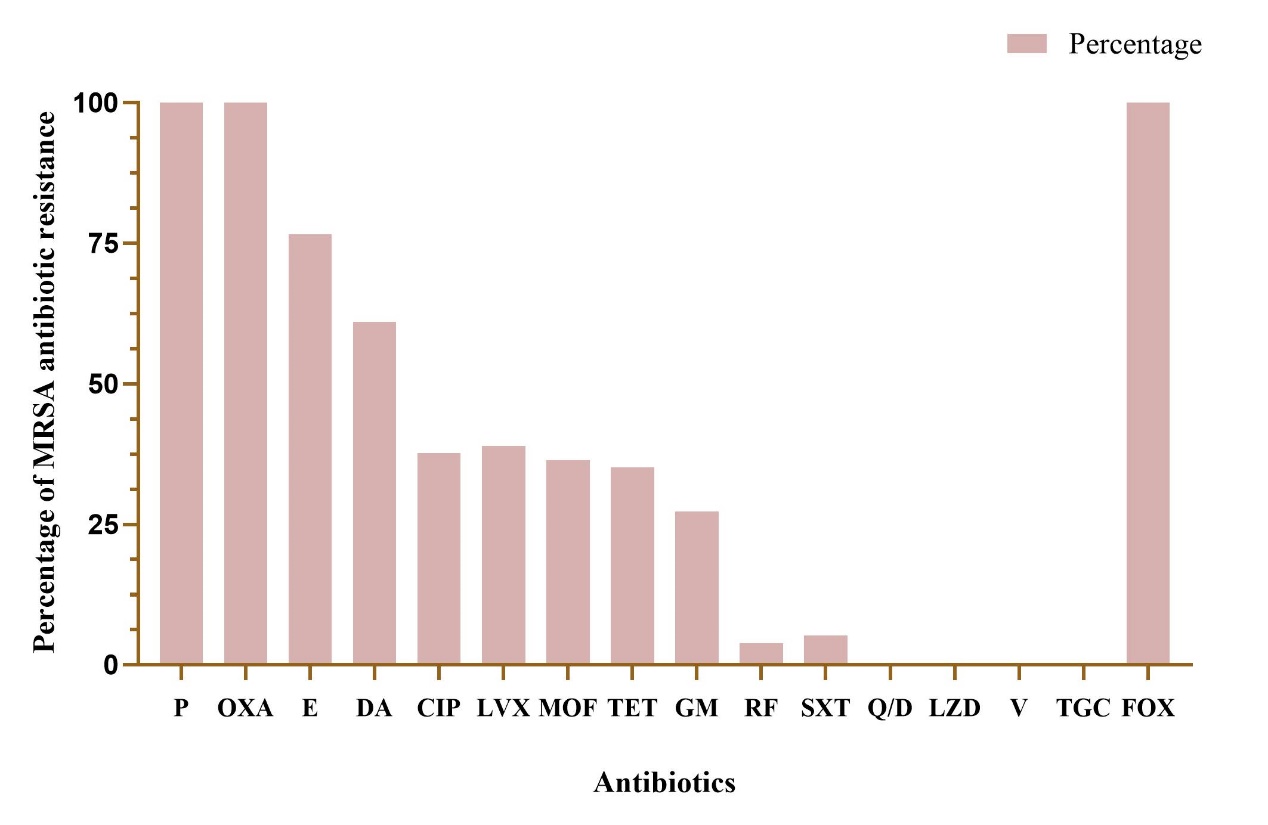
**

**Supplementary Figure 1.** Antimicrobial resistance profiles of MRSA isolates from adult and pediatric patients. Abbreviations: P, penicillin; OXA, oxacillin; E, erythromycin; DA, clindamycin; CIP, ciprofloxacin; LVX, levofloxacin; MOF, moxifloxacin; TET, tetracycline; GM, gentamicin; RF, rifampicin; SXT, trimethoprim-sulfamethoxazole; Q/D, quinupristin/dalfopristin; LZD, linezolid; V, vancomycin; TGC, tigecycline; FOX, cefoxitin.
